# *Pseudobactrodesmium* (Dactylosporaceae, Eurotiomycetes, Fungi) a Novel Lignicolous Genus

**DOI:** 10.3389/fmicb.2020.00456

**Published:** 2020-04-02

**Authors:** Wei Dong, Kevin D. Hyde, Mingkwan Doilom, Xian-Dong Yu, D. Jayarama Bhat, Rajesh Jeewon, Saranyaphat Boonmee, Gen-Nuo Wang, Sarunya Nalumpang, Huang Zhang

**Affiliations:** ^1^Faculty of Agriculture and Food, Kunming University of Science and Technology, Kunming, China; ^2^Department of Entomology and Plant Pathology, Faculty of Agriculture, Chiang Mai University, Chiang Mai, Thailand; ^3^Center of Excellence in Fungal Research, Mae Fah Luang University, Chiang Rai, Thailand; ^4^Key Laboratory for Plant Diversity and Biogeography of East Asia, Kunming Institute of Botany, Chinese Academy of Sciences, Kunming, China; ^5^World Agroforestry Centre, East and Central Asia, Kunming, China; ^6^Retired, Curca, India; ^7^Department of Health Sciences, Faculty of Science, University of Mauritius, Reduit, Mauritius; ^8^Faculty of Environmental Science and Engineering, Kunming University of Science and Technology, Kunming, China; ^9^Department of Botany, University of British Columbia, Vancouver, BC, Canada; ^10^Yunnan Key Lab of Soil Carbon Sequestration and Pollution Control, Kunming University of Science and Technology, Kunming, China

**Keywords:** *Bactrodesmium*, multi-gene, sheath, submerged wood, taxonomy

## Abstract

During our ongoing surveys of fungi on submerged wood in the Greater Mekong Subregion, we collected two new species similar to *Bactrodesmium longisporum*. *Pseudobactrodesmium* gen. nov. is introduced to accommodate the new species, *P. aquaticum*, *P. chiangmaiensis* and *B. longisporum* is transferred to this genus. Fasciculate conidiophores, enteroblastic conidiogenous cells and subulate to fusiform, phragmoseptate conidia with a tapering apical cell and sheath characterize the genus. *Pseudobactrodesmium aquaticum* has longer conidia than *P. chiangmaiensis*. The placement of *Pseudobactrodesmium* in *Dactylosporaceae* (Eurotiomycetes) is a novel finding based on analyses of combined LSU, SSU, ITS and RPB2 sequence data. Our study reveals that *Pseudobactrodesmium* is likely to be a speciose genus with different species in streams around the world.

## Introduction

*Dactylosporaceae* accommodates ecologically and morphologically diverse genera, and was reinstated by Diederich et al. (2018) to replace *Sclerococcaceae* (Réblová et al., 2016). For example, the freshwater genus *Cylindroconidiis* has holoblastic conidiogenous cells (Yu et al., 2018), while the terrestrial genera *Pseudosclerococcum* and *Rhopalophora* are apothecial ascomycetes and dematiaceous phialidic hyphomycetes, respectively (Réblová et al., 2016; Olariaga et al., 2019). The terrestrial and marine genus *Sclerococcum* (= *Dactylospora*) has loose sporodochia with catenate conidia or apothecia-like ascomata often growing on lichens or decaying wood (Hawksworth, 1975; Jones et al., 1999; Pang et al., 2014; Pino-Bodas et al., 2017). Additionally, *Fusichalara minuta*, which is a dematiaceous phialidic hyphomycete, and some beetle-associated strains also cluster in this family (Vargasasensio et al., 2014; Tedersoo et al., 2017).

Aquatic hyphomycetes are a morphologically diverse and polyphyletic group (Shenoy et al., 2006; Baschien et al., 2013; Su et al., 2016). Species with similar morphological characters are difficult to identify without molecular data. Previously, identification was mostly carried out based on morphology and only a few asexual taxa have been subjected to phylogenetic studies (Goh and Hyde, 1996; Cai et al., 2002; Cai and Hyde, 2007). With more molecular data becoming available for phylogenetic analyses, numerous new combinations have been proposed to accommodate poorly documented hyphomycetous species (Lu et al., 2018; Yang et al., 2018a, b). Molecular data also demonstrated that some previously known congeneric species are now distributed in different families, e.g., *Monodictys arctica* in *Leptosphaeriaceae* (Day et al., 2006), *M. capensis* in *Pleomonodictydaceae* (Hernández-Restrepo et al., 2017), and some other *Monodictys* species in *Parabambusicolaceae* (Tanaka et al., 2015). Although the polyphyletic nature of some hyphomycetous genera were partially resolved, e.g., *Dendryphion*, *Sporidesmium* and torula-like species (Su et al., 2016), fresh collections with molecular data are still needed to obtain a natural classification of hyphomycetes.

Invalidly established by Berkeley and Broome (1865) with *Sporidesmium abruptum* as the type, the hyphomycetous genus *Bactrodesmium* was segregated from *Sporidesmium*, with *B. abruptum* as the lectotype (Hughes, 1958). *Bactrodesmium* is distributed worldwide with more than 48 species (Wijayawardene et al., 2017a; Index Fungorum database^1^). It was regarded as a member of Dothideomycetes based on the sexual-asexual morph connection between *Bactrodesmium obliquum* and *Stuartella suttonii* (Funk and Shoemaker, 1983; Wijayawardene et al., 2017b). However, with molecular evidence, *Bactrodesmium* was shown to be polyphyletic, as *B. gabretae* clustered within Helotiales, Leotiomycetes (Koukol and Kolárová, 2010), *B. cubense* had affinities to *Morosphaeriaceae*, Dothideomycetes (Hernández-Restrepo et al., 2017) and *B. pallidum* clustered in *Savoryellaceae*, Sordariomycetes (Hernández-Restrepo et al., 2017). Recently, *Bactrodesmium fasciculare* was transferred to a newly established genus *Pleotrichocladium* in *Melanommataceae* (Dothideomycetes) based on molecular data and morphology (Hernández-Restrepo et al., 2017). Moreover, the generic type *B. abruptum* was tentatively placed in Dothideomycetes based on morphological evidence but molecular data is still lacking (Pem et al., 2019). The phylogenetic position of other species still needs to be investigated.

We are studying the freshwater fungi on submerged wood along a north–south latitudinal gradient in the Asian/Australian region (Hyde et al., 2016) and have published several papers on the Greater Mekong Subregion ([citeskum]BR90,BR88,BR87,BR89,BR85,BR86[citeekum]Zhang et al., 2011, 2012, 2013, 2014, 2016, 2017; Dong et al., 2018; Wei et al., 2018; Yu et al., 2018; Wang et al., 2019). In this study, two taxa morphologically similar to *Bactrodesmium longisporum* were collected from submerged wood. To clarify the classification of the two new collections, we analyzed a combined LSU, SSU, ITS and RPB2 sequence dataset and compared their morphological characters. *Pseudobactrodesmium*, a new genus with two new species, and one new combination are introduced. Morphologically similar genera are compared with *Pseudobactrodesmium* and the taxonomic placements of *Bactrodesmium* species are discussed.

## Materials and Methods

### Isolation and Morphology

The decaying wood samples were collected from freshwater streams in Chiang Mai Province, Thailand and Yunnan Province, China. Specimens were placed in zip-lock plastic bags with moist cotton or tissue paper and taken to the laboratory. Morphological observations were carried out after incubation at room temperature for 1–2 weeks. Colonies were examined using a Nikon SMZ-171 dissecting microscope. Photomicrographs were made with a Nikon ECLIPSE Ni compound microscope fitted with a Canon EOS 600D digital camera. Measurements were made with the Tarosoft (R) Image Frame Work program. Images used for figures were processed with Adobe Photoshop CS5 software (Adobe Systems, United States). Single spore isolations were made from conidia onto potato dextrose agar (PDA) at room temperature, as detailed in Chomnunti et al. (2014) and cultured as outlined by Vijaykrishna et al. (2004) and Liu et al. (2010). Herbarium specimens (dry wood with fungal material) were deposited in the herbarium of Mae Fah Luang University (MFLU), Chiang Rai, Thailand and herbarium of Cryptogams, Kunming Institute of Botany Academia Sinica (HKAS), Kunming, China. Living cultures were deposited in Mae Fah Luang University Culture Collection (MFLUCC) and Kunming Institute of Botany Culture Collection (KUMCC). Facesoffungi and Index Fungorum numbers were registered as in Jayasiri et al. (2015) and Index Fungorum (2020), respectively.

### DNA Extraction, PCR Amplification and Sequencing

Fungi were grown on PDA for 20–30 days at 25°C. A Biospin Fungus Genomic DNA Extraction Kit (Bioer Technology Co., Hangzhou, China) was used to extract total genomic DNA from fresh mycelia according to the manufacturer’s instructions. DNA amplification was performed by polymerase chain reaction (PCR). LSU, SSU, ITS and RPB2 gene regions were amplified using the primer pairs LR0R/LR5, NS1/NS4, ITS5/ITS4 and RPB2-5F/RPB2-7cR, respectively (Vilgalys and Hester, 1990; White et al., 1990; Rehner and Samuels, 1994; Liu et al., 1999). The amplifications were carried out in a 25 μL reaction volume containing 9.5 μL ddH_2_O, 12.5 μL 2 × PCR Master Mix, 1 μL DNA template, 1 μL each primer (10 μM). The PCR thermal cycles for the amplification of the gene regions followed the methods in Jeewon et al. (2004); Réblová et al. (2011), and Su et al. (2015). PCR products were checked on 1% agarose electrophoresis gels stained with Gel Red. The sequencing reactions were carried out by Shanghai Sangon Biological Engineering Technology and Services Co., Shanghai, China.

### Phylogenetic Analyses

The qualities of raw sequences generated in this study were checked with Finch TV version 1.4.0. Based on nucleotide BLAST^2^ and previous publications (Raja et al., 2008; Koukol and Kolárová, 2010; Réblová et al., 2012, 2016; Diederich et al., 2013; Pang et al., 2014; Boonmee et al., 2016; Su et al., 2016; Yang et al., 2016; Hernández-Restrepo et al., 2017; Pino-Bodas et al., 2017; Yu et al., 2018; Dayarathne et al., 2019; Ekanayaka et al., 2019; Olariaga et al., 2019), related sequences together with newly generated ones were selected for constructing a phylogenetic tree. All sequences used in this study are listed in Table 1. The individual datasets of LSU, SSU, ITS and RPB2 were aligned using MAFFT v. 7.409 online version (Kazutaka and Standley, 2016) and manually verified with BioEdit v.7.2.5 Biological Sequence Alignment Editor (Ibis BioSciences, CA). Phylogenetic analyses of the combined dataset (LSU, SSU, ITS and RPB2) were inferred with maximum likelihood (ML) and Bayesian inference (BI) analyses.

**TABLE 1:** Taxa used in the phylogenetic analysis and their corresponding GenBank accession numbers.

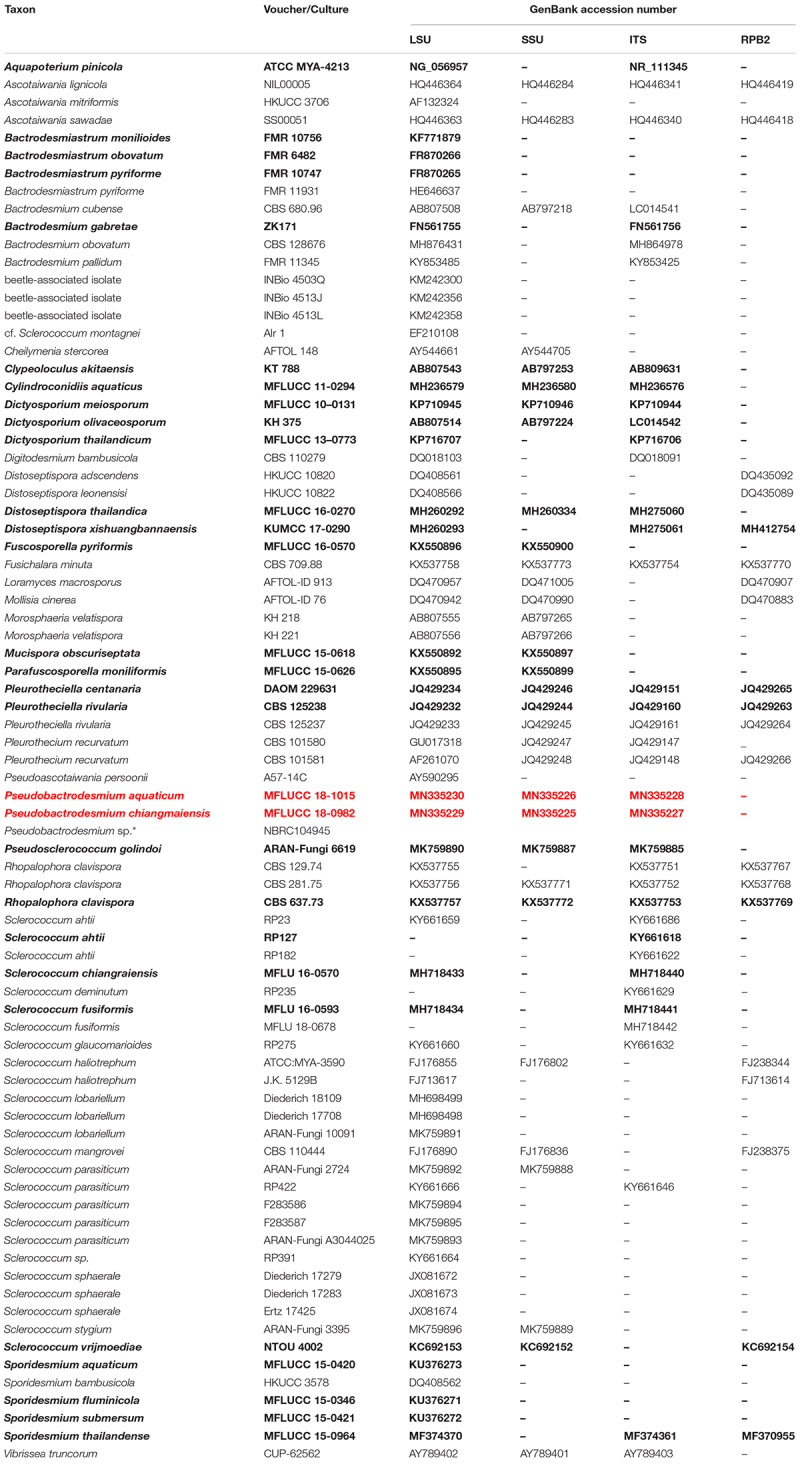

Ex-type strains are in bold; newly generated sequences are highlighted in red. *The LSU, SSU, and ITS sequences of *Pseudobactrodesmium* sp. NBRC 104945 are available online at https://www.nite.go.jp/nbrc/catalogue/NBRCCatalogueDetailServlet?ID=NBRC&CAT=00104945.

**FIGURE 1:**
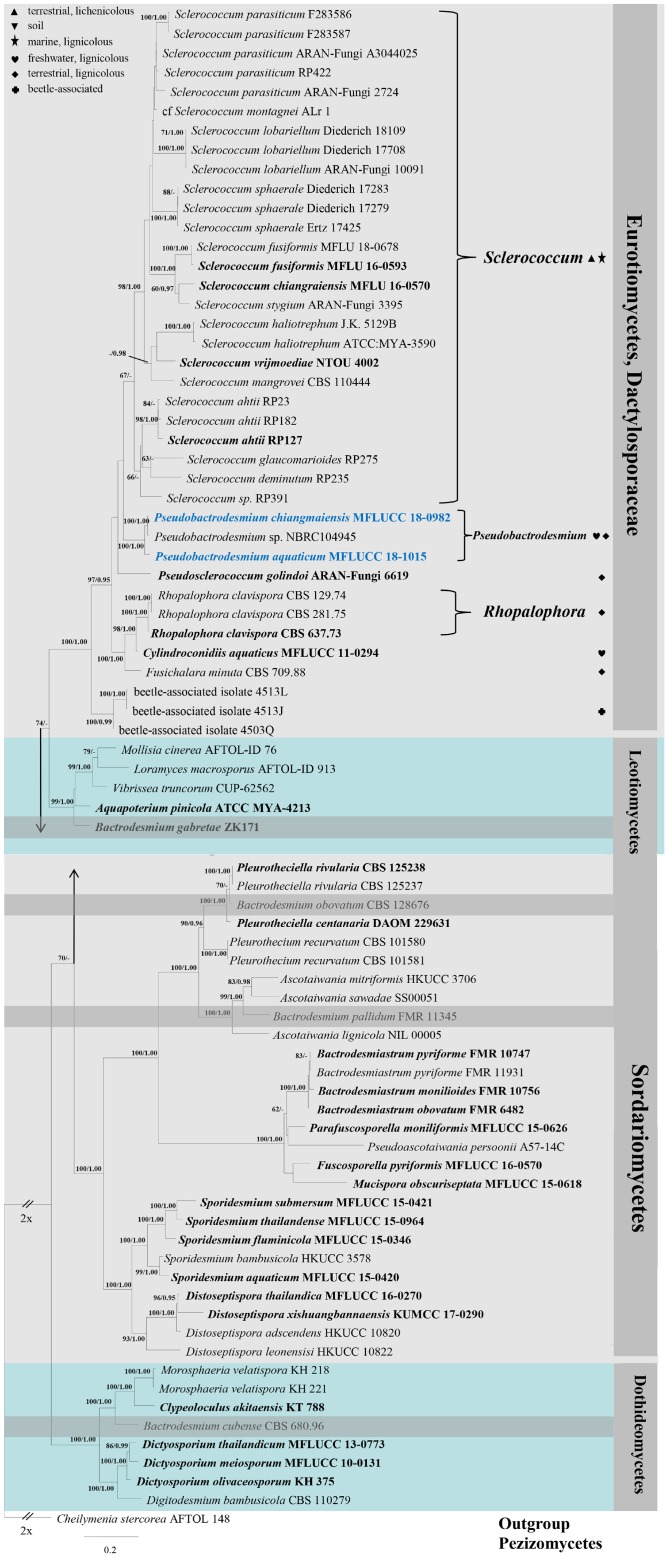
RAxML tree generated from combined LSU, SSU, ITS, and RPB2 sequence data. Bootstrap support values for maximum likelihood (the first value) equal to or greater than 60% and Bayesian posterior probabilities (the second value) equal to or greater than 0.95 are given above or below the nodes. The tree is rooted to *Cheilymenia stercorea* (AFTOL 148) (Pezizomycetes). The ex-type strains are indicated in bold and newly generated sequences are indicated in blue. Four bactrodesmium-like species are highlighted in gray background. Symbols after generic names in Eurotiomycetes indicate the habitats of taxa as explained in the phylogram.

A ML analysis was performed with RAxML-HPC v.8 on XSEDE in CIPRES Science Gateway (Miller et al., 2010, 2015) with 1000 rapid bootstrap replicates. The model selected for ML was GTRGAMMA. Maximum likelihood bootstrap values equal to or greater than 60% are given above or below the nodes (first value, Figure 1). Bayesian inference was conducted with MrBayes v. 3.1.2 (Huelsenbeck and Ronquist, 2001) to evaluate posterior probabilities (BPP) (Rannala and Yang, 1996) by Markov chain Monte Carlo (MCMC) sampling. The best-fit model was GTR + I + G for LSU, SSU and RPB2, and SYM + I + G for ITS. Six simultaneous Markov chains were run for one million generations and trees were sampled every 100 generation (resulting in 10000 trees). The first 2500 trees, representing the burn-in phase of the analyses, were discarded and the remaining 7500 trees were used for calculating posterior probabilities (PP) in the majority rule consensus tree (Larget and Simon, 1999). Bayesian posterior probabilities (BPP) equal to or greater than 0.95 are given above or below the nodes (second value, Figure 1).

Phylogenetic trees were viewed with FigTree v1.4.0^3^ ^,4^ and edited using Microsoft Office PowerPoint 2007 (Microsoft Corporation, WA, United States). The new sequences were deposited in GenBank (Table 1).

## Results

### Phylogenetic Analyses

Combined LSU, SSU, ITS, and RPB2 gene regions were employed to explore the taxonomy of new collections. The alignment comprised 79 strains (including two new strains) with an alignment length of 4381 total characters. The RAxML analysis resulted in a best scoring likelihood tree selected with a final value for the combined dataset ln L = −39819.188166. The matrix has 2564 distinct alignment patterns, with 62% of undetermined characters or gaps. Estimated base frequencies are as follows: A = 0.256432, C = 0.229269, G = 0.279558, T = 0.234740; substitution rates AC = 1.300864, AG = 2.616909, AT = 1.328213, CG = 1.028043, CT = 6.168383, GT = 1.000000; gamma distribution shape parameter α = 0.360140.

In the phylogenetic tree (Figure 1), the two new isolates are shown in Eurotiomycetes and distantly related to *Bactrodesmium cubense* (Dothideomycetes), *B. gabretae* (Leotiomycetes), and *B. obovatum* and *B. pallidum* (Sordariomycetes). The morphologically similar genera, e.g., *Bactrodesmiastrum*, *Dictyosporium*, *Digitodesmium*, *Distoseptispora*, and *Sporidesmium*, have phylogenetically unrelated relationships with our new strains (Figure 1). *Pseudobactrodesmium aquaticum* and *P. chiangmaiensis* constitute a distinct clade in the family *Dactylosporaceae* (Figure 1).

### Taxonomy

***Pseudobactrodesmium*** H. Zhang, W. Dong & K. D. Hyde, **gen. nov.**

*Index Fungorum number*: IF557247; *Facesoffungi number*: FoF07525

*Etymology*: in reference to bactrodesmium-like morphology

*Saprobic* on submerged wood in freshwater or decaying wood in terrestrial habitats. **Sexual morph:** Undetermined. **Asexual morph:** Hyphomycetous. *Colonies* sporodochial, superficial, effuse, gregarious or scattered, brown, punctiform. *Mycelium* mostly immersed, composed of septate, branched, hyaline hyphae. *Conidiophores* macronematous, mononematous, fasciculate, compact, erect, subcylindrical, septate, usually unbranched, brown, smooth. *Conidiogenous cells* enteroblastic, with inconspicuous proliferations, integrated, terminal, subcylindrical, pale brown. *Conidia* acrogenous, solitary, dry, thin-walled, smooth-walled, clavate, subcylindrical, narrowly fusiform or subulate, euseptate, phragmoseptate, brown, often enveloped by a hyaline, spherical sheath at the apex. *Apical cells* elongated, tapering gradually toward the apex, with globose tuberculate apex.

*Type species*: *Pseudobactrodesmium aquaticum* W. Dong, H. Zhang & K.D. Hyde

*Notes*: *Pseudobactrodesmium* is characterized by enteroblastic conidiogenous cells and subulate or fusiform, evenly pigmented conidia with a tapering apical cell. In contrast, *Bactrodesmium* as typified by *B. abruptum* is quite distinct in producing holoblastic conidiogenous cells and clavate to fusiform, unevenly colored conidia which are mid or dark brown at the upper part and becoming paler toward the basal cell, 3- to multi-transversely septate, with very dark bands at the septa, the upper one thick and black, unequal cells, the penultimate cell much longer than any of the others (Ellis, 1971). *Pseudobactrodesmium* nests well within the family *Dactylosporaceae* in our phylogenetic tree of the combined sequence dataset (Figure 1). The unique combination of morphological characters of *Pseudobactrodesmium* stands apart from other existing genera in this family (Hawksworth, 1975; Ellis, 1976; Jones et al., 1999; Réblová et al., 2016; Yu et al., 2018; Olariaga et al., 2019). *Pseudobactrodesmium* is morphologically similar to a few dematiaceous hyphomycetous genera with long or short, septate conidia, e.g., *Bactrodesmiastrum* (Holubová-Jechová, 1984), *Bactrodesmium* (Ellis, 1971, 1976), *Digitodesmium* (Boonmee et al., 2016), *Distoseptispora* (Su et al., 2016), *Scolecostigmina* (Braun et al., 1999), and *Sporidesmium* (Su et al., 2016), but they are separated by molecular evidence (Figure 1).

**FIGURE 2:**
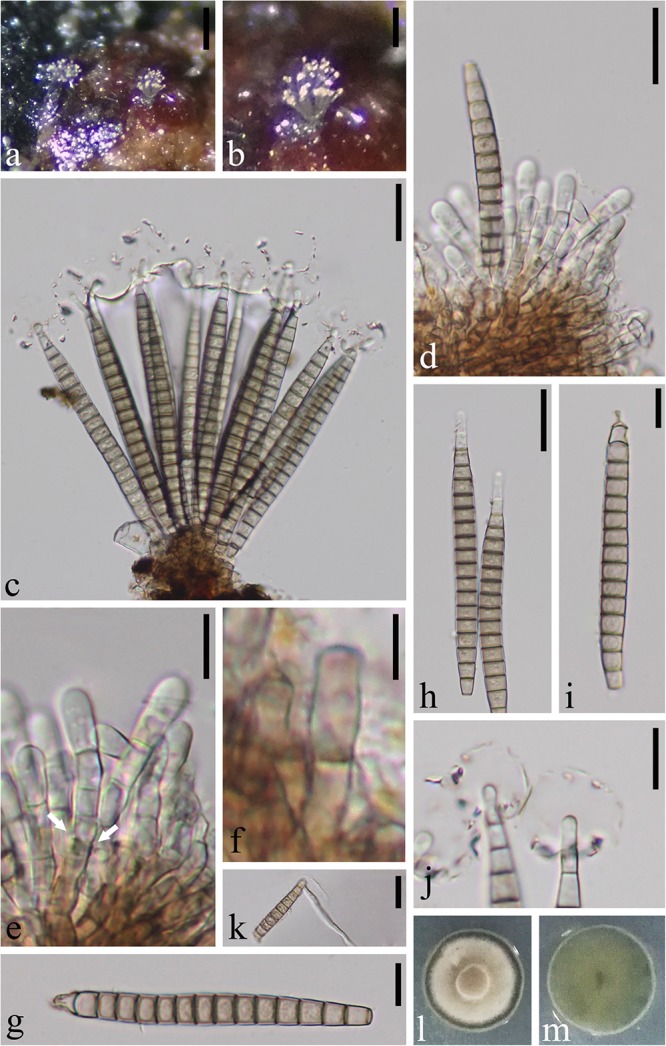
*Pseudobactrodesmium aquaticum* (MFLU 18-1171, holotype). **(a,b)** Colonies on submerged wood. **(c,d)** Conidiophores bearing conidia. **(e)** Enteroblastic conidiogenous cells (arrow). **(f)** Conidiophore. **(g**–**i)** Conidia. **(j)** Conidial tips with sheaths. **(k)** Germinated conidium. **(l)** Colony on PDA (front view). **(m)** Colony on PDA (bottom view). Scale bars: **(a)** 200 μm, **(b)** 100 μm, **(c,d,h,k)** 20 μm, **(e,g,i,j)** 10 μm, **(f)** 5 μm.

***Pseudobactrodesmium aquaticum*** W. Dong, H. Zhang & K.D. Hyde, **sp. nov.,** Figure 2

*Index Fungorum number*: IF557248; *Facesoffungi number*: FoF07526

*Etymology*: aquaticum in reference to the aquatic habitat

*Holotype*: MFLU 18-1171

*Saprobic* on submerged wood in freshwater. **Sexual morph:** Undetermined. **Asexual morph:** Hyphomycetous. *Colonies* sporodochial, superficial, effuse, gregarious or scattered, brown, punctiform. *Mycelium* mostly immersed, composed of septate, branched, hyaline hyphae. *Conidiophores* 26–38 × 3–4.5 μm ($\bar{x}$ = 34 × 3.8 μm, *n* = 10), macronematous, mononematous, fasciculate, compact, erect, subcylindrical, the apex slightly wider than the base, septate, slightly constricted at septa, usually unbranched, brown, smooth. *Conidiogenous cells* enteroblastic, with inconspicuous proliferations, integrated, terminal, subcylindrical, pale brown. *Conidia* (80–)90–105 × 6–8.5 μm ($\bar{x}$ = 95 × 7.5 μm, *n* = 20), acrogenous, solitary, dry, thin-walled, smooth-walled, clavate when young, subcylindrical to narrowly fusiform, or subulate when mature, straight or slightly curved, euseptate, (15–)16–19-phragmoseptate, slightly constricted and darker at septa, pale brown to brown, obscurely guttulate, wedge-shaped at basal cell, with tapering apical cells, often enveloped by a hyaline, spherical, thin, gelatinous sheath at the apex, 13–20 μm diam. *Apical cells* elongated, up to 6 μm long, tapering gradually toward the apex, easily becoming senescent, subhyaline, with obscured, subglobose tuberculate ends.

*Culture characteristics*: On PDA, colony circular, slow growing, reaching 10 mm in 50 days at 25°C, gray to brown from above, dark gray from below, surface rough, dry, raised, entire at edge.

*Material examined*: CHINA, Yunnan Province, Pingbian City, on submerged wood in a stream, 20 September 2017, W. Dong, WF-24A-1 (MFLU 18-1171, **holotype**), ex-type living culture MFLUCC 18-1015; *ibid.* WF-24A-2 (HKAS 101707, **isotype**), ex-isotype living culture KUMCC 18-0056.

*Notes*: *Pseudobactrodesmium aquaticum* is introduced as the type species of *Pseudobactrodesmium* having the typical euseptate, phragmoseptate conidia with apical sheath. The enteroblastic conidiogenous cells are particularly obvious in Figure 2e.

**FIGURE 3:**
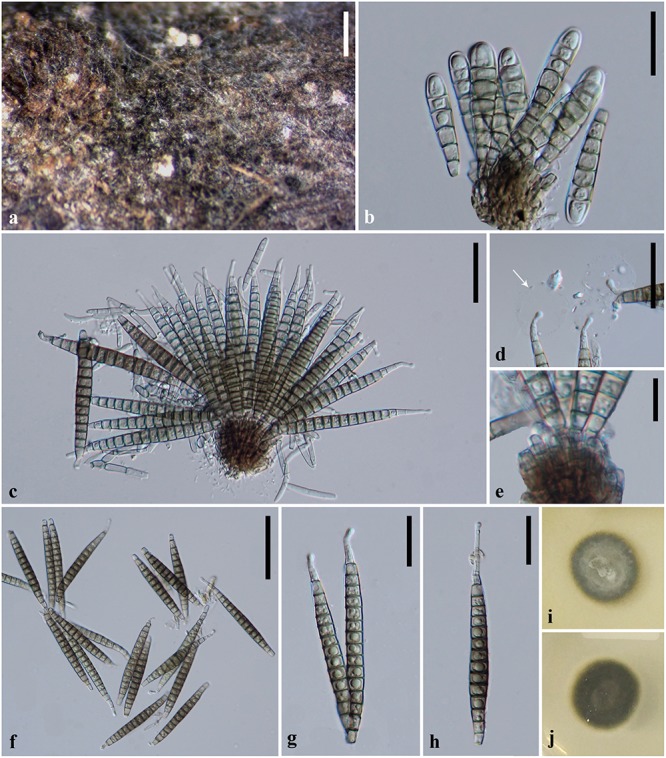
*Pseudobactrodesmium chiangmaiensis* (MFLU 18-0994, holotype). **(a)** Colonies on submerged wood. **(b,c)** Conidiophores bearing conidia. **(d)** Conidial tips with sheaths (arrow). **(e)** Apex of conidiophores. **(f**–**h)** Conidia. **(i)** Colony on PDA (front view). **(j)** Colony on PDA (bottom view). Scale bars: **(a)** 500 μm, **(b,g,h)** 20 μm, **(c,d)** 30 μm, **(e)** 10 μm, **(f)** 50 μm.

***Pseudobactrodesmium chiangmaiensis*** X. D. Yu, W. Dong & K. D. Hyde, **sp. nov.,** Figure 3

*Index Fungorum number*: IF557249; *Facesoffungi number*: FoF07527

*Etymology*: name reflects Chiang Mai, from where the species was collected

*Holotype*: MFLU 18-0994

*Saprobic* on submerged wood in freshwater. **Sexual morph:** Undetermined. **Asexual morph:** Hyphomycetous. *Colonies* sporodochial, superficial, effuse, gregarious or scattered, dark brown to black, punctiform. *Mycelium* mostly immersed, composed of septate, branched, hyaline hyphae. *Conidiophores* 15–23 × 2.5–4 μm ($\bar{x}$ = 21.5 × 3.5 μm, *n* = 10), macronematous, mononematous, fasciculate, compact, erect, subcylindrical, septate, slightly constricted at the septa, usually unbranched, brown, smooth. *Conidiogenous cells* enteroblastic, with inconspicuous proliferations, integrated, terminal, subcylindrical, pale brown. *Conidia* 40–90 × 5.5–8.5 μm ($\bar{x}$ = 70 × 7 μm, *n* = 50), acrogenous, solitary, dry, thin-walled, smooth-walled, clavate when young, subcylindrical to narrowly fusiform, or subulate when mature, straight or slightly curved, euseptate, 6–19-phragmoseptate, slightly constricted and darker at septa, pale brown to brown, obscurely guttulate, wedge-shaped at basal cell, with tapering apical cells, often enveloped by a hyaline, spherical, thin, gelatinous sheath at the apex, 17–21 μm diam. *Apical cells* elongated, up to but rarely 16 μm long, tapering gradually toward apex, subhyaline, with subglobose tuberculate ends.

*Culture characteristics*: On PDA, colony circular, reaching 15 mm in 20 days at 25°C, dark gray to dark brown from above, dark gray to black from below, surface rough, dry, raised, margin entire.

*Material examined*: Thailand, Chiang Mai Province, on submerged wood in a stream, 9 February 2018, X.D. Yu, Y11 (MFLU 18-0994, **holotype**), ex-type living culture MFLUCC 18-0982.

*Notes*: *Pseudobactrodesmium chiangmaiensis* differs from *P. aquaticum* in having shorter conidia (40–90 × 5.5–8.5 μm vs. (80–)90–105 × 6–8.5 μm), longer apical cells (up to 16 μm vs. up to 6 μm), as well as darker colonies on the host (dark brown to black vs. brown). The conidial sheaths are obscure in *P. chiangmaiensis* when mounted in water, while they are easily observed in *P. aquaticum*. This is probably because the specimens were senescent which led the sheaths to deliquesce. In our phylogenetic tree, *P. chiangmaiensis* groups with *P. aquaticum* with strong bootstrap support (100% MLBS, 1.00 PP, Figure 1). However, a comparison of sequence data between *P. chiangmaiensis* and *P. aquaticum* shows a difference of 6, 7, 20, and 32 nucleotides in LSU, SSU, ITS, and TEF gene regions, respectively. This indicates that they are distinct species according to guidelines of Jeewon and Hyde (2016).

***Pseudobactrodesmium longisporum*** (M.B. Ellis) W. Dong & K.D. Hyde, **comb. nov.**

*Index Fungorum number*: IF557250; *Facesoffungi number*: FoF07466

≡ *Bactrodesmium longisporum* M.B. Ellis, More Dematiaceous Hyphomycetes (Kew): 68 (1976)

≡ *Stigmina longispora* (M.B. Ellis) S. Hughes, N. Z. Jl Bot. 16(3): 353 (1978)

= *Bactrodesmium stilboideum* R. F. Castañeda & G. R. W. Arnold, Revta Jardín bot. Nac., Univ. Habana 6(1): 48 (1985)

≡ *Stigmina longispora* var. *stilboidea* (R. F. Castañeda & G. R. W. Arnold) J. Mena & Mercado, Reporte de Investigacion del Instituto de Ecología y Sistemática, Academia de Ciencias de Cuba, Ser. Bot. 17: 10 (1987)

*Holotype*: On dead wood of *Alnus* sp. in Great Britain (IMI 63746 B)

*Known distribution*: New Zealand (Hughes, 1978), Australia (Vijaykrishna and Hyde, 2006), Brazil (Barbosa and Gusmão, 2011; Barbosa et al., 2013; Santa Izabel and Gusmão, 2016, 2018), Cuba (Castañeda Ruiz and Arnold, 1985), Great Britain (Ellis, 1976), Hong Kong, China (Wong and Hyde, 2001), India (Prabhugaonkar, 2011), Venezuela (Castañeda Ruiz et al., 2009), México (Heredia et al., 2018), Peru (Shearer et al., 2015), Philippines (Cai et al., 2003), South Africa (Hyde et al., 1998), Thailand (Hu et al., 2010; this study), United States (Raja et al., 2007).

*Notes*: *Bactrodesmium longisporum* was described by Ellis (1976) with a line-drawing. It was subsequently synonymized with *Stigmina longispora* by Hughes (1978) who observed percurrently proliferating conidiophores in old specimens from New Zealand. *Bactrodesmium stilboideum* is another synonym listed in Index Fungorum database. However, they can be distinguished by the aggregation of conidiophores (synnematous in *B. stilboideum* vs. mononematous, fasciculate conidiophores in *B. longisporum*) (Ellis, 1976; Castañeda Ruiz and Arnold, 1985).

A Thai strain of *B. longisporum* (NBRC 104945) clustered with *Pseudobactrodesmium chiangmaiensis* (MFLUCC 18-0982) in our phylogenetic tree (Figure 1). A comparison of sequence data between NBRC 104945 and MFLUCC 18-0982 shows a difference of 2, 281, 5 nucleotides in LSU, SSU and ITS gene regions, respectively (NBRC 104945 has 3 major insertions spanning over 281 nucleotides in SSU gene). In this study, we name NBRC 104945 as *Pseudobactrodesmium* sp. until its morphological characters are established to formally name this isolate. Five additional strains with only ITS2 sequence data are named as *Bactrodesmium longisporum* in GenBank. However, their status should be treated with caution as they represent OTUS from a metagenomic study of a heap leaching system (Hu et al., 2015) and further evidence of conspecificity is needed.

Unfortunately, the holotype specimen of *B. longisporum* (IMI 63746 B), does not exist in herbarium IMI.^5^ According to protologue description of the holotype (Ellis, 1976), *B. longisporum* (IMI 63746 B) has similar conidial size to *P. chiangmaiensis* (MFLU 18-0994) (50–80 × 7–8 μm in former vs. 40–90 × 5.5–8.5 μm in latter). However, *P. chiangmaiensis* has elongated apical cells (up to 16 μm long) with subglobose tuberculate ends, which were not described and drawn in protologue of *B. longisporum* (Ellis, 1976). The conidiophores of *B. longisporum* are up to 50 μm long, but only 15–23 μm long in *P. chiangmaiensis*. The size of apical sheath of *B. longisporum* is also unclear. Thus, we treat them as different species and synonymize *B. longisporum* under *Pseudobactrodesmium* as the third species in the genus. Epitypification of *Pseudobactrodesmium longisporum* is needed using a collection from its type locality.

## Discussion

*Bactrodesmium longisporum* has been recorded as having a worldwide distribution, however these records have not been verified with molecular data. The type of *B. longisporum* also appears to be lost and therefore its identity cannot be verified. We therefore designate our new species of *Pseudobactrodesmium* from China as the generic type, describe a second species from Thailand and transfer *Bactrodesmium longisporum* to the new genus. However, it is likely that many collections of this species have been misidentified and as more collections are made from different countries, we would expect *Pseudobactrodesmium* to become speciose.

*Bactrodesmium* is a complex genus in need of extensive taxonomic reassessment. Pem et al. (2019) reviewed the holotype material of *Bactrodesmium abruptum* (≡ *Sporidesmium abruptum*) and tentatively placed the generic type in Dothideomycetes *incertae sedis* based on morphology. Both *B. cubense* and *B. obovatum* produce clavate or obovate conidia with darker septa and unequal cells, similar to the type species *B. abruptum* (Ellis, 1971; Zucconi and Lunghini, 1997). However, our phylogenetic study shows that they belong to different classes, Dothideomycetes and Sordariomycetes, respectively (Figure 1). *Bactrodesmium gabretae* differs from *B. abruptum* by its transversely or occasionally oblique, distoseptate conidia, and phylogenetically clustered in Leotiomycetes (Figure 1). *Bactrodesmium pallidum* is different from *B. abruptum* but similar to our new genus *Pseudobactrodesmium* in conidial shape (Ellis, 1959), and phylogeny places this species in Sordariomycetes (Figure 1). Our phylogenetic study is in agreement with the studies of Koukol and Kolárová (2010) and Hernández-Restrepo et al. (2017).

Although molecular data of *B. abruptum* is still missing, the working hypothesis of *Bactrodesmium sensu stricto* in Dothideomycetes provides further evidence for the introduction of *Pseudobactrodesmium*. *Pseudobactrodesmium* shares some morphological characters with *Digitodesmium* in having acrogenous, long, transversely septate conidia with a hyaline sheath at the apex (Kirk, 1981; Boonmee et al., 2016). However, the semi-macronematous, moniliform conidiophores and digitate conidia of *Digitodesmium* are clearly distinguishable from the macronematous, subcylindrical conidiophores and subcylindrical to narrowly fusiform, or subulate conidia of *Pseudobactrodesmium*. Phylogeny also segregates them into different classes, viz. *Pseudobactrodesmium* in Eurotiomycetes, and *Digitodesmium* in Dothideomycetes (Tsui et al., 2006; Boonmee et al., 2016; this study). The conidia of *Scolecostigmina* are superficially similar to those of *Pseudobactrodesmium*, but the former is characterized by conspicuously annellate conidiogenous cells, thick-walled, smooth to verrucose conidia occasionally with a few longitudinal or oblique septa or a few intermixed distosepta, contrasting with inconspicuously proliferating conidiogenous cells and thin-walled, smooth, transversely phragmoseptate conidia with a hyaline, spherical sheath at the apex in *Pseudobactrodesmium* (Braun et al., 1999; Crous et al., 2013). *Scolecostigmina*, typified by *S. mangiferae*, clustered in Capnodiales (Dothideomycetes) (Crous et al., 2013), while *Pseudobactrodesmium* clustered in *Dactylosporaceae* (Eurotiomycetes). *Pseudobactrodesmium longisporum* is superficially similar to *Gangliostilbe malabarica* in the conidial shape and apical sheath, but the synnemata and apically rounded conidia of the latter can easily be separated from the former (Xia et al., 2015). These characters of *G. malabarica* are also distinguished from those in the collection of Castañeda Ruiz and Arnold (1985) bearing the name *Bactrodesmium stilboideum*.

It is challenging to reconstruct the phylogeny of *Bactrodesmium* considering lack of living cultures of *B. abruptum*. The species having clavate or obovate, long or short, transversely septate conidia with or without apical sheath are common and scattered in different groups (Ellis, 1976; Hughes, 1978; Holubová-Jechová, 1984; Braun et al., 1999; Koukol and Kolárová, 2010; Crous et al., 2013; Xia et al., 2015; Boonmee et al., 2016; Su et al., 2016; Hernández-Restrepo et al., 2017; Videira et al., 2017). These groups of fungi are morphologically similar and therefore molecular characters are of crucial importance to clarify their taxonomy. The sequence data of *B. abruptum* is needed in the future to clarify the natural classification of *Bactrodesmium*.

## Data Availability Statement

The datasets generated for this study can be found in the NCBI GenBank: MN335230, MN335226, MN335228, MN335229, MN335225, and MN335227.

## Author Contributions

WD conducted the experiments, analyzed the data, and wrote the manuscript. KH planned the experiments. MD analyzed the data. X-DY conducted the experiments. DB and RJ revised the manuscript. SB funded the experiments. G-NW conducted the experiments. SN planned the experiments. HZ planned the experiments, analyzed the data, and wrote the manuscript. All authors revised the manuscript.

## Conflict of Interest

The authors declare that the research was conducted in the absence of any commercial or financial relationships that could be construed as a potential conflict of interest.

## Footnotes

1 http://www.indexfungorum.org/names/Names.asp

2 http://www.ncbi.nlm.nih.gov/

3 http://tree.bio.ed.ac.uk/

4 http://jebl.sourceforge.net/

5 http://www.herbimi.info/herbimi/results.htm?name=Bactrodesmium%20longisporum

## References

[B1] Barbosa F. Gusmão L. (2011). Conidial fungi from semi-arid Caatinga biome of Brazil. Rare freshwater hyphomycetes and other new records. *Mycosphere* 2 475–485.

[B2] Barbosa F. R. Raja H. A. Shearer C. A. Gusmão L. F. P. (2013). Some freshwater fungi from the Brazilian semi-arid region, including two new species of hyphomycetes. *Cryptogam. Mycol.* 34 243–258. 10.7872/crym.v34.iss2.2013.243

[B3] Baschien C. Tsui C. K. M. Gulis V. Szewzyk U. Marvanová L. (2013). The molecular phylogeny of aquatic hyphomycetes with affinity to the leotiomycetes. *Fungal Biol.* 117 660–672. 10.1016/j.funbio.2013.07.004 24012305

[B4] Berkeley M. J. Broome C. E. (1865). Notices of British fungi (1038–1062). *Ann. Mag. Nat. Hist.* 15 400–404.

[B5] Boonmee S. D’souza M. J. Luo Z. L. Pinruan U. Tanaka K. Su H. Y. (2016). *Dictyosporiaceae* fam. nov. *Fungal Divers.* 80 457–482. 10.1007/s13225-016-0363-z

[B6] Braun U. Mouchacca J. McKenzie E. (1999). Cercosporoid hyphomycetes from New Caledonia and some other South Pacific islands. *N.Z. J. Bot.* 37 297–327. 10.1080/0028825X.1999.9512636

[B7] Cai L. Hyde K. D. (2007). Anamorphic fungi from freshwater habitats in China: *Dictyosporium tetrasporum* and *Exserticlava yunnanensis* spp. nov., and two new records for *Pseudofuscophialis lignicola* and *Pseudobotrytis terrestris*. *Mycoscience* 48 290–296. 10.1007/S10267-007-0369-1

[B8] Cai L. Zhang K. Q. McKenzie E. H. C. Ho W. H. Hyde K. D. (2002). *Acrodictys liputii* sp. nov. and *Digitodesmium bambusicola* sp. nov. from bamboo submerged in the Liput River in the Philippines. *Nova Hedwig.* 75 525–532. 10.1127/0029-5035/2002/0075-0525

[B9] Cai L. Zhang K. Q. McKenzie E. H. C. Hyde K. D. (2003). Freshwater fungi from bamboo and wood submerged in the Liput River in the Philippines. *Fungal Divers.* 13 1–12.

[B10] Castañeda Ruiz R. F. Arnold G. R. (1985). Deuteromycotina de Cuba. I. Hyphomycetes. *Revista Jard. Bot. Nac.* 6 47–67.

[B11] Castañeda Ruiz R. F. Guerrero B. Adamo G. M. Morillo O. Minter D. W. Stadler M. (2009). A new species of *Selenosporella* and two microfungi recorded from a cloud forest in Mérida, Venezuela. *Mycotaxon* 109 63–74. 10.5248/109.63

[B12] Chomnunti P. Hongsanan S. Aguirre-Hudson B. Tian Q. Peršoh D. Dhami M. K. (2014). The sooty moulds. *Fungal Divers.* 66 1–36. 10.1007/s13225-014-0278-5

[B13] Crous P. W. Braun U. Hunter G. C. Wingfield M. J. Verkley G. Shin H.-D. (2013). Phylogenetic lineages in *Pseudocercospora*. *Stud. Mycol.* 75 37–114. 10.3114/sim0005 24014898 PMC3713886

[B14] Day M. J. Gibas C. F. C. Fujimura K. E. Egger K. N. Currah R. S. (2006). *Monodictys arctica*, a new hyphomycete from the roots of *Saxifraga oppositifolia* collected in the Canadian High Arctic. *Mycotaxon* 98 261–272. 10.7939/R3CF9J63X

[B15] Dayarathne M. C. Maharachchikumbura S. S. N. Jones E. B. G. Dong W. Devadatha B. Yang J. (2019). Phylogenetic revision of *Savoryellaceae* and evidence for its ranking as a subclass. *Front. Microbiol.* 10:840. 10.3389/fmicb.2019.00840 31133992 PMC6514050

[B16] Diederich P. Ertz D. Lawrey J. D. Sikaroodi M. Untereiner W. A. (2013). Molecular data place the hyphomycetous lichenicolous genus *Sclerococcum* close to *Dactylospora* (Eurotiomycetes) and *S. parmeliae* in *Cladophialophora* (Chaetothyriales). *Fungal Divers.* 58 61–72. 10.1007/s13225-012-0179-4

[B17] Diederich P. Lawrey J. D. Ertz D. (2018). The 2018 classification and checklist of lichenicolous fungi, with 2000 non-lichenized, obligately lichenicolous taxa. *Bryologist* 121 340–425. 10.1639/0007-2745-121.3.340

[B18] Dong W. Hyde K. D. Bhat D. J. Zhang H. (2018). Introducing *Aculeata aquatica* gen. et sp. nov., *Minimelanolocus thailandensis* sp. nov. and *Thysanorea aquatica* sp. nov. (*Herpotrichiellaceae*, Chaetothyriales) from freshwater in northern Thailand. *Mycol. Prog.* 17 617–629. 10.1007/s11557-018-1389-2

[B19] Ekanayaka A. H. Jones E. B. G. Hyde K. D. Zhao Q. (2019). A stable phylogeny for *Dactylosporaceae*. *Cryptogam. Mycol.* 40 23–44. 10.5252/cryptogamie-mycologie2019v40a3

[B20] Ellis M. B. (1959). Clasterosporium and some allied Dematiaceae-phragmosporae. II. *Mycol. Pap.* 72 1–75.

[B21] Ellis M. B. (1971). *Dematiaceous Hyphomycetes.* Kew: Commonwealth Mycological Institute.

[B22] Ellis M. B. (1976). *More Dematiaceous Hyphomycetes.* Kew: Commonwealth Mycological Institute.

[B23] Funk A. Shoemaker R. (1983). *Stuartella suttonii* n. sp., the teleomorph of *Bactrodesmium obliquum* var. *suttonii*. *Can. J. Bot.* 61 2277–2279. 10.1139/b83-249

[B24] Goh T. K. Hyde K. D. (1996). *Cryptophiale multiseptata*, sp. nov. from submerged wood in Australia, and keys to the genus. *Mycol. Res.* 100 999–1004. 10.1016/S0953-7562(96)80054-2

[B25] Hawksworth D. L. (1975). A revision of lichenicolous fungi accepted by Keissler in *Coniothecium*. *Trans. Br. Mycol. Soc.* 65 219–238. 10.1016/S0007-1536(75)80005-2

[B26] Heredia G. Arias-Mota R. M. Mena-Portales J. Castañeda-Ruiz R. F. (2018). Saprophytic synnematous microfungi. New records and known species for Mexico. *Rev. Mex. Biodivers.* 89 604–618. 10.22201/ib.20078706e.2018.3.2352

[B27] Hernández-Restrepo M. Gené J. Castañeda-Ruiz R. F. Mena-Portales J. Crous P. W. Guarro J. (2017). Phylogeny of saprobic microfungi from Southern Europe. *Stud. Mycol.* 86 53–97. 10.1016/j.simyco.2017.05.002 28626275 PMC5470572

[B28] Holubová-Jechová V. (1984). *Bactrodesmiastrum*, a new genus of lignicolous Hyphomycetes. *Folia Geobot. Phytotx.* 19 103–106. 10.1007/BF02853338

[B29] Hu D. M. Cai L. Chen H. Bahkali A. H. Hyde K. D. (2010). Fungal diversity on submerged wood in a tropical stream and an artificial lake. *Biodivers. Conserv.* 19 3799–3808. 10.1007/s10531-010-9927-5

[B30] Hu Q. Guo X. Liang Y. L. Hao X. D. Ma L. Y. Yin H. Q. (2015). Comparative metagenomics reveals microbial community differentiation in a biological heap leaching system. *Res. Microbiol.* 166 525–534. 10.1016/j.resmic.2015.06.005 26117598

[B31] Huelsenbeck J. P. Ronquist F. (2001). MRBAYES: Bayesian inference of phylogenetic trees. *Bioinformatics* 17 754–755. 10.1093/bioinformatics/17.8.754 11524383

[B32] Hughes S. J. (1958). Revisiones hyphomycetum aliquot cum appendice de nominibus rejiciendis. *Can. J. Bot.* 36 727–836. 10.1139/b58-067

[B33] Hughes S. J. (1978). New Zealand fungi 25. Miscellaneous species. *N.Z. J. Bot.* 16 311–370. 10.1080/0028825X.1978.10425143

[B34] Hyde K. D. Fryar S. Tian Q. Bahkali A. H. Xu J. C. (2016). Lignicolous freshwater fungi along a north–south latitudinal gradient in the Asian/Australian region; can we predict the impact of global warming on biodiversity and function? *Fungal Ecol.* 19 190–200. 10.1016/j.funeco.2015.07.002

[B35] Hyde K. D. Goh T. K. Steinke T. D. (1998). Fungi on submerged wood in the Palmiet River, Durban, South Africa. *S. Afr. J. Bot.* 64 151–162. PMID:NOPMID

[B36] Index Fungorum (2020). Available online at: http://www.indexfungorum.org/names/names.asp (accessed January, 2020).

[B37] Jayasiri S. C. Hyde K. D. Ariyawansa H. A. Bhat J. Buyck B. Cai L. (2015). The Faces of fungi database: fungal names linked with morphology, phylogeny and human impacts. *Fungal Divers.* 74 3–18. 10.1007/s13225-015-0351-8

[B38] Jeewon R. Hyde K. D. (2016). Establishing species boundaries and new taxa among fungi: recommendations to resolve taxonomic ambiguities. *Mycosphere* 7 1669–1677. 10.5943/mycosphere/7/11/4

[B39] Jeewon R. Liew E. C. Y. Hyde K. D. (2004). Phylogenetic evaluation of species nomenclature of *Pestalotiopsis* in relation to host association. *Fungal Divers.* 17 39–55.

[B40] Jones E. B. G. Abdel-Wahab M. A. Alias S. A. Hsieh S. Y. (1999). *Dactylospora mangrovei* sp. nov. (Discomycetes, ascomycota) from mangrove wood. *Mycoscience* 40 317–320. 10.1007/BF02463875

[B41] Kazutaka K. Standley D. M. (2016). A simple method to control over-alignment in the MAFFT multiple sequence alignment program. *Bioinformatics* 32 1933–1942. 10.1093/bioinformatics/btw108 27153688 PMC4920119

[B42] Kirk P. (1981). New or interesting microfungi II. Dematiaceous hyphomycetes from Esher Common, Surrey. *Trans. Br. Mycol. Soc.* 77 279–297. 10.1016/S0007-1536(81)80031-9

[B43] Koukol O. Kolárová Z. (2010). *Bactrodesmium gabretae* (anamorphic Helotiales), a new sporodochial species described from spruce needles. *Nova Hedwig.* 91 243–248. 10.1127/0029-5035/2010/0091-0243

[B44] Larget B. Simon D. L. (1999). Markov chain Monte Carlo algorithms for the Bayesian analysis of phylogenetic trees. *Mol. Biol. Evol.* 16 750–759. 10.1093/oxfordjournals.molbev.a026160

[B45] Liu A.-R. Chen S.-C. Wu S.-Y. Xu T. Guo L.-D. Jeewon R. (2010). Cultural studies coupled with DNA based sequence analyses and its implication on pigmentation as a phylogenetic marker in *Pestalotiopsis* taxonomy. *Mol. Phylogenet. Evol.* 57 528–535. 10.1016/j.ympev.2010.07.017 20692352

[B46] Liu Y. J. Whelen S. Hall B. D. (1999). Phylogenetic relationships among ascomycetes: evidence from an RNA polymerse II subunit. *Mol. Biol. Evol.* 16 1799–1808. 10.1093/oxfordjournals.molbev.a026092 10605121

[B47] Lu Y.-Z. Liu J.-K. Hyde K. D. Jeewon R. Kang J.-C. Fan C. (2018). A taxonomic reassessment of tubeufiales based on multi-locus phylogeny and morphology. *Fungal Divers.* 92 131–344. 10.1007/s13225-018-0411-y

[B48] Miller M. A. Pfeiffer W. Schwartz T. (2010). “Creating the CIPRES science gateway for inference of large phylogenetic trees,” in *Proceedings of the Gateway Computing Environments Workshop (GCE)* (New Orleans, LA: IEEE), 1–8.

[B49] Miller M. A. Schwartz T. Pickett B. E. He S. Klem E. B. Scheuermann R. H. (2015). A RESTful API for access to phylogenetic tools via the CIPRES science gateway. *Evol. Bioinform.* 11 43–48. 10.4137/EBO.S21501 25861210 PMC4362911

[B50] Olariaga I. Teres J. Martín J. Prieto M. Baral H.-O. (2019). *Pseudosclerococcum golindoi* gen. et sp. nov., a new taxon with apothecial ascomata and a *Chalara*-like anamorph within the Sclerococcales (Eurotiomycetes). *Mycol. Prog.* 18 895–905. 10.1007/s11557-019-01500-7

[B51] Pang K. L. Guo S. Y. Alias S. A. Hafellner J. Jones E. B. G. (2014). A new species of marine *Dactylospora* and its phylogenetic affinities within the Eurotiomycetes, Ascomycota. *Bot. Mar.* 57 315–321. 10.1515/bot-2014-0025

[B52] Pem D. Jeewon R. Bhat D. J. Doilom M. Boonmee S. Hongsanan S. (2019). Mycosphere notes 275-324: a morpho-taxonomic revision and typification of obscure Dothideomycetes genera (*incertae sedis*). *Mycosphere* 10 1115–1246. 10.5943/mycosphere/10/1/22

[B53] Pino-Bodas R. Zhurbenko M. P. Stenroos S. (2017). Phylogenetic placement within *Lecanoromycetes* of lichenicolous fungi associated with *Cladonia* and some other genera. *Persoonia* 39 91–117. 10.3767/persoonia.2017.39.05 29503472 PMC5832959

[B54] Prabhugaonkar A. (2011). *Studies on Diversity and Activity of Microfungi Associated with Indigenous Palms of Western Ghats, India.* Ph.D. thesis, Goa University, Goa].

[B55] Raja H. A. Miller A. N. Shearer C. A. (2008). Freshwater ascomycetes: *Aquapoterium pinicola*, a new genus and species of Helotiales (Leotiomycetes) from Florida. *Mycologia* 100 141–148. 10.1080/15572536.2008.11832506 18488360

[B56] Raja H. A. Stchigel A. M. Miller A. N. Crane J. L. Shearer C. A. (2007). Hyphomycetes from the Great Smoky Mountains National Park, including three new species. *Fungal Divers.* 26 271–286.

[B57] Rannala B. Yang Z. (1996). Probability distribution of molecular evolutionary trees: a new method of phylogenetic inference. *J. Mol. Evol.* 43 304–311. 10.1007/BF02338839 8703097

[B58] Réblová M. Gams W. Seifert K. A. (2011). *Monilochaetes* and allied genera of the Glomerellales, and a reconsideration of families in the Microascales. *Stud. Mycol.* 68 163–191. 10.3114/sim.2011.68.07 21523193 PMC3065989

[B59] Réblová M. Seifert K. A. Fournier J. Stepánek V. (2012). Phylogenetic classification of *Pleurothecium* and *Pleurotheciella* gen. nov. and its dactylaria-like anamorph (Sordariomycetes) based on nuclear ribosomal and protein-coding genes. *Mycologia* 104 1299–1314. 10.3852/12-035 22684295

[B60] Réblová M. Untereiner W. A. Štìpánek V. Gams W. (2016). Disentangling *Phialophora* section *Catenulatae*: disposition of taxa with pigmented conidiophores and recognition of a new subclass, Sclerococcomycetidae (Eurotiomycetes). *Mycol. Prog.* 16 27–46. 10.1007/s11557-016-1248-y

[B61] Rehner S. A. Samuels G. J. (1994). Taxonomy and phylogeny of *Gliocladium* analysed from nuclear large subunit ribosomal DNA sequences. *Mycol. Res.* 98 625–634. 10.1016/S0953-7562(09)80409-7

[B62] Santa Izabel T. d. S. Gusmão L. F. P. (2016). Fungal succession on plant debris in three humid forests enclaves in the Caatinga biome of Brazil. *Braz. J. Bot.* 39 1065–1076. 10.1007/s40415-016-0305-8

[B63] Santa Izabel T. d. S. Gusmão L. F. P. (2018). Richness and diversity of conidial fungi associated with plant debris in three enclaves of Atlantic Forest in the Caatinga biome of Brazil. *Plant Ecol. Evol.* 151 35–47. 10.5091/plecevo.2018.1332

[B64] Shearer C. A. Zelski S. E. Raja H. A. Schmit J. P. Miller A. N. Janovec J. P. (2015). Distributional patterns of freshwater ascomycetes communities along an Andes to Amazon elevational gradient in Peru. *Biodivers. Conserv.* 24 1877–1897. 10.1007/s10531-015-0911-y

[B65] Shenoy B. D. Jeewon R. Wu W. P. Bhat D. J. Hyde K. D. (2006). Ribosomal and RPB2 DNA sequence analyses suggest that *Sporidesmium* and morphologically similar genera are polyphyletic. *Mycol. Res.* 110 916–928. 10.1016/j.mycres.2006.06.004 16908125

[B66] Su H. Y. Hyde K. D. Maharachchikumbura S. S. N. Ariyawansa H. A. Luo Z. L. Promputtha I. (2016). The families *Distoseptisporaceae* fam. nov., *Kirschsteiniotheliaceae*, *Sporormiaceae* and *Torulaceae*, with new species from freshwater in Yunnan Province, China. *Fungal Divers.* 80 375–409. 10.1007/s13225-016-0362-0

[B67] Su H. Y. Udayanga D. Luo Z. L. Manamgoda D. S. Zhao Y. C. Yang J. (2015). Hyphomycetes from aquatic habitats in Southern China: species of *Curvularia* (*Pleosporaceae*) and *Phragmocephala* (*Melannomataceae*). *Phytotaxa* 226 201–216. 10.11646/phytotaxa.226.3.1

[B68] Tanaka K. Hirayama K. Yonezawa H. Sato G. Toriyabe A. Kudo H. (2015). Revision of the Massarineae (Pleosporales, Dothideomycetes). *Stud. Mycol.* 82 75–136. 10.1016/j.simyco.2015.10.002 26955201 PMC4774272

[B69] Tedersoo L. Bahram M. Puusepp R. Nilsson R. H. James T. Y. (2017). Novel soil-inhabiting clades fill gaps in the fungal tree of life. *Microbiome* 5:42. 10.1186/s40168-017-0259-5 28388929 PMC5385062

[B70] Tsui C. K. M. Berbee M. L. Jeewon R. Hyde K. D. (2006). Molecular phylogeny of *Dictyosporium* and allied genera inferred from ribosomal DNA. *Fungal Divers.* 21 157–166.

[B71] Vargasasensio G. Pintotomas A. Rivera B. Hernandez M. Hernandez C. Sotomontero S. (2014). Uncovering the cultivable microbial diversity of Costa Rican beetles and its ability to break down plant cell wall components. *PLoS One* 9:e113303. 10.1371/journal.pone.0113303 25411842 PMC4239062

[B72] Videira S. Groenewald J. Nakashima C. Braun U. Barreto R. W. de Wit P. J. (2017). Mycosphaerellaceae–chaos or clarity? *Stud. Mycol.* 87 257–421. 10.1016/j.simyco.2017.09.003 29180830 PMC5693839

[B73] Vijaykrishna D. Hyde K. D. (2006). Inter- and intra stream variation of lignicolous freshwater fungi in tropical Australia. *Fungal Divers.* 21 203–224.

[B74] Vijaykrishna D. Mostert L. Jeewon R. Gams W. Hyde K. D. Crous P. W. (2004). *Pleurostomophora*, an anamorph of *Pleurostoma* (Calosphaeriales), a new anamorph genus morphologically similar to *Phialophora*. *Stud. Mycol.* 50 387–395.

[B75] Vilgalys R. Hester M. (1990). Rapid genetic identification and mapping of enzymatically amplified ribosomal DNA from several *Cryptococcus* species. *J. Bacteriol.* 172 4238–4246. 10.1128/jb.172.8.4238-4246.1990 2376561 PMC213247

[B76] Wang G.-N. Yu X.-D. Dong W. Bhat D. J. Boonmee S. Zhang D. (2019). Freshwater hyphomycetes in Eurotiomycetes: a new species of *Minimelanolocus* and a new collection of *Thysanorea papuana* (*Herpotrichiellaceae*). *Mycol. Prog.* 18 511–522. 10.1007/s11557-019-01473-7

[B77] Wei M. J. Zhang H. Dong W. Boonmee S. Zhang D. (2018). Introducing *Dictyochaeta aquatica* sp. nov. and two new species of *Chloridium* (*Chaetosphaeriaceae*, Sordariomycetes) from aquatic habitats. *Phytotaxa* 362 187–199. 10.11646/phytotaxa.362.2.5

[B78] White T. J. Bruns T. Lee S. Taylor J. (1990). “Amplification and direct sequencing of fungal ribosomal RNA genes for phylogenetics,” in *PCR Protocols: A Guide to Methods and Applications*, eds Innis M. A. Gelfand D. H. Sninsky J. J. White T. J. (New York: Academic Press), 315–322. 10.1016/b978-0-12-372180-8.50042-1

[B79] Wijayawardene N. N. Hyde K. D. Rajeshkumar K. C. Hawksworth D. L. Madrid H. Kirk P. M. (2017a). Notes for genera: Ascomycota. *Fungal Divers.* 86 1–594. 10.1007/s13225-017-0386-0

[B80] Wijayawardene N. N. Hyde K. D. Tibpromma S. Wanasinghe D. N. Thambugala K. M. Tian Q. (2017b). Towards incorporating asexual fungi in a natural classification: checklist and notes 2012–2016. *Mycosphere* 8 1457–1555. 10.5943/mycosphere/8/9/10

[B81] Wong M. K. M. Hyde K. D. (2001). Diversity of fungi on six species of *Gramineae* and one species of *Cyperaceae* in Hong Kong. *Mycol. Res.* 105 1485–1491. 10.1017/s0953756201004695

[B82] Xia J.-W. Ma Y.-R. Gao J.-M. Li Z. Zhang X.-G. (2015). *Sporidesmiopsis malloti* sp. nov. and new records from southern China. *Mycotaxon* 130 827–833. 10.5248/130.827

[B83] Yang J. Liu J. K. Hyde K. D. Jones E. B. G. Liu Z. Y. (2018a). New species in *Dictyosporium*, new combinations in *Dictyocheirospora* and an updated backbone tree for *Dictyosporiaceae*. *Mycokeys* 36 83–105. 10.3897/mycokeys.36.27051 30057482 PMC6060225

[B84] Yang J. Liu N. G. Liu J. K. Hyde K. D. Jones E. B. G. Liu Z. Y. (2018b). Phylogenetic placement of *Cryptophiale*, *Cryptophialoidea*, *Nawawia*, *Neonawawia* gen. nov. and *Phialosporostilbe*. *Mycosphere* 9 1132–1150. 10.5943/mycosphere/9/6/5

[B85] Yang J. Maharachchikumbura S. S. N. Bhat D. J. Hyde K. D. Mckenzie E. H. C. Jones E. B. G. (2016). Fuscosporellales, a new order of aquatic and terrestrial hypocreomycetidae (Sordariomycetes). *Cryptogam. Mycol.* 4 449–475. 10.7872/crym/v37.iss4.2016.1 26240444

[B86] Yu X. D. Dong W. Bhat D. J. Boonmee S. Zhang D. Zhang H. (2018). *Cylindroconidiis aquaticus* gen. et sp. nov., a new, lineage of aquatic hyphomycetes in *Sclerococcaceae* (Eurotiornycetes). *Phytotaxa* 372 79–87. 10.11646/phytotaxa.372.1.6

[B87] Zhang H. Dong W. Hyde K. D. Bahkali A. H. Liu J. K. Zhou D. Q. (2016). Molecular data shows *Didymella aptrootii* is a new genus in *Bambusicolaceae*. *Phytotaxa* 247 99–108. 10.11646/phytotaxa.247.2.1

[B88] Zhang H. Dong W. Hyde K. D. Maharachchikumbura S. S. N. Hongsanan S. Jayarama Bhat D. (2017). Towards a natural classification of Annulatascaceae-like taxa: introducing Atractosporales ord. nov. and six new families. *Fungal Divers.* 85 75–110. 10.1007/s13225-017-0387-z

[B89] Zhang H. Hyde K. D. Abdel-Wahab M. A. Abdel-Aziz F. A. Ariyawansa H. A. Ko T. W. K. (2013). A modern concept for *Helicascus* with a Pleurophomopsis-like asexual state. *Sydowia* 65 147–166.

[B90] Zhang H. Hyde K. D. Mckenzie E. H. C. Bahkali A. H. Zhou D. Q. (2012). Sequence data reveals phylogenetic affinities of *Acrocalymma aquatica* sp. nov., *Aquasubmersa mircensis* gen. et sp. nov. and *Clohesyomyces aquaticus* (freshwater coelomycetes). *Cryptogam. Mycol.* 33 333–346. 10.7872/crym.v33.iss3.2012.333

[B91] Zhang H. Hyde K. D. Zhao Y. C. McKENZIE E. H. C. Zhou D. Q. (2014). Freshwater ascomycetes: *Lophiostoma vaginatispora* comb. nov. (Dothideomycetes, Pleosporales, *Lophiostomaceae*) based on morphological and molecular data. *Phytotaxa* 176 184–191. 10.11646/phytotaxa.176.1.18

[B92] Zhang H. Jones E. B. G. Zhou D. Q. Bahkali A. H. Hyde K. D. (2011). Checklist of freshwater fungi in Thailand. *Cryptogam. Mycol.* 32 199–217. 10.7872/crym.v32.iss2.2011.199

[B93] Zucconi L. Lunghini D. (1997). Studies on Mediterranean hyphomycetes. VI. Remarks on *Bactrodesmium*, and *B. cubense* comb. nov. *Mycotaxon* 63 323–328.

